# GluA2-Lacking AMPA Receptors and Nitric Oxide Signaling Gate Spike-Timing–Dependent Potentiation of Glutamate Synapses in the Dorsal Raphe Nucleus

**DOI:** 10.1523/ENEURO.0116-17.2017

**Published:** 2017-06-02

**Authors:** Samir Haj-Dahmane, Jean Claude Béïque, Roh-Yu Shen

**Affiliations:** 1 Research Institute on Addictions, University at Buffalo, Buffalo, NY; 2 Department of Pharmacology and Toxicology, University at Buffalo, Buffalo, NY; 3 Department of Cellular and Molecular Medicine Faculty of Medicine, University of Ottawa, Ottawa, Ontario Canada

**Keywords:** AMPA, dorsal raphe, LTP, nitric oxide, NMDA, serotonin

## Abstract

The dorsal raphe nucleus (DRn) receives glutamatergic inputs from numerous brain areas that control the function of DRn serotonin (5-HT) neurons. By integrating these synaptic inputs, 5-HT neurons modulate a plethora of behaviors and physiological functions. However, it remains unknown whether the excitatory inputs onto DRn 5-HT neurons can undergo activity-dependent change of strength, as well as the mechanisms that control their plasticity. Here, we describe a novel form of spike-timing–dependent long-term potentiation (tLTP) of glutamate synapses onto rat DRn 5-HT neurons. This form of synaptic plasticity is initiated by an increase in postsynaptic intracellular calcium but is maintained by a persistent increase in the probability of glutamate release. The tLTP of glutamate synapses onto DRn 5-HT is independent of NMDA receptors but requires the activation of calcium-permeable AMPA receptors and voltage-dependent calcium channels. The presynaptic expression of the tLTP is mediated by the retrograde messenger nitric oxide (NO) and activation of cGMP/PKG pathways. Collectively, these results indicate that glutamate synapses in the DRn undergo activity-dependent synaptic plasticity gated by NO signaling and unravel a previously unsuspected role of NO in controlling synaptic function and plasticity in the DRn.

## Significance Statement

Glutamatergic inputs to DRn 5-HT neurons are involved in the regulation of numerous physiologic functions and behavior tasks that require associative learning. However, it remains unknown whether glutamate synapses onto DRn 5-HT neurons can undergo activity-dependent changes in strength. Here, we describe a novel form of spike-timing–dependent LTP in the DRn gated by the activation of calcium-permeable AMPA receptors, voltage-dependent calcium channels, and NO signaling. This form of plasticity may represent a cellular mechanism by which 5-HT neurons can regulate associative learning. These data also unravel the role of NO in controlling synaptic function and plasticity in the DRn.

## Introduction

In the mammalian brain, neurons containing 5-hydroxytryptamine (5-HT; serotonin) are clustered in small nuclei, called raphe nuclei, located in the brainstem (Dahlström and Fuxe, 1964). Among these nuclei, the dorsal raphe nucleus (DRn) is the largest, containing more than half of the total 5-HT neurons found in the brain (Descarries et al. 1982). These neurons provide extensive and widespread efferent projections to various targets (Imai et al. 1986), thereby controlling the function of neuronal networks distributed throughout the entire brain (Jacobs and Azmitia, 1992). Consistent with its widespread innervation, DRn 5-HT neurons have been implicated in a plethora of functions, including arousal (Monti, 2011), feeding (Voigt and Fink, 2015), aggression (Bortolato et al. 2013), sensorimotor functions (Jacobs and Fornal, 1997), and reward and emotional behaviors (Cools et al. 2008). In addition, dysfunction of the central 5-HT system is involved in the pathophysiology of autism, schizophrenia, depression, and anxiety (Abi-Dargham et al. 1997).

Given the diverse physiologic and pathophysiological roles of DRn 5-HT neurons, numerous studies have focused on determining precise neuronal circuits and the mechanisms that regulate the function of these neurons. It is well established that the DRn receives inputs from remarkably diverse brain areas that use various neurotransmitters (Peyron et al. 1998; Lee et al. 2005), including glutamate (Pollak Dorocic et al. 2014; Weissbourd et al. 2014). Most of the glutamatergic inputs to the DRn originate from cortical and subcortical regions and from raphe nuclei (Kalen et al. 1985; Lee et al. 2003). These inputs form an extensive network that has synapses to both 5-HT and non–5-HT neurons (Weissbourd et al. 2014; Geddes et al. 2016), and they regulate the overall activity of DRn 5-HT neurons. By integrating these various glutamatergic inputs, DRn 5-HT neurons modulate specific behaviors and regulate numerous physiologic functions. As such, determining the mechanisms that regulate the strength and plasticity of glutamate synapses onto DRn 5-HT neurons is essential for understanding the role of the 5-HT system in controlling various behaviors and physiologic functions. Although the regulation of the strength of glutamate synapses onto DRn 5HT neurons by various neurotransmitters, including 5-HT (Geddes et al. 2015), noradrenaline (Haj-Dahmane and Shen, 2014), neuropeptides, and endocannabinoids (Liu et al. 2002; Haj-Dahmane and Shen, 2005, 2009), has been extensively studied, it remains unknown whether these synapses undergo activity-dependent synaptic plasticity. Here, we show that glutamate synapses onto DRn 5-HT neurons exhibit spike-timing–dependent potentiation. This form of Hebbian plasticity is NMDA receptor independent and involves the activation of calcium-permeable GluA2-lacking AMPA receptors (AMPARs) and nitric oxide (NO) signaling pathways. As such, the results of this study unravel an important role of GluA2-lacking AMPARs and NO signaling in controlling synaptic plasticity in the DRn and, hence, the function of DRn 5-HT neurons.

## Materials and Methods

### Brain slice preparation

All the experimental procedures in the present study were approved by the University at Buffalo Animal Care and Use Committee and were in accordance with the National Institutes of Health Guidelines for the Care and Use of Laboratory Animals. Brain slices containing the DRn were prepared from 6- to 8-week-old male Sprague-Dawley rats (Envigo) using previously described procedures (Haj-Dahmane, 2001). In brief, rats were anesthetized with isoflurane and killed by decapitation. A block of brainstem area containing the DRn was isolated, and coronal slices (300–350 µm) were cut using a vibratome (Lancer series 1000; Leica Biosystems) in ice-cold modified Ringer’s solution of the following composition (in mm): 110 choline-Cl; 2.5 KCl; 0.5 CaCl_2_; 7 MgSO_4_; 1.25 NaH_2_PO_4_; 26.2 NaHCO_3_; 11.6 sodium l-ascorbate; 3.1 sodium pyruvate; and 25 glucose, equilibrated with 95% O_2_/5% CO_2_. Slices were incubated for 45 min at 35°C and then at room temperature for at least 1 h in a holding chamber containing regular Ringer’s solution (in mm): 119 NaCl; 2.5 CaCl_2_; 1.3 MgSO_4_; 1 NaH_2_PO_4_; 26.2 NaHCO_3_; and 11 glucose, continuously bubbled with a mixture of 95% O_2_/5% CO_2_. After recovery, slices were transferred to a recording chamber (Warner Instruments) mounted on a fixed upright microscope and continuously perfused (2–3 mL/min) with Ringer’s solution saturated with 95% O_2_/5% CO_2_ and heated to 30 ± 1°C using a solution heater (Warner Instruments).

### Electrophysiological recordings

DRn neurons were visualized using a BX 51 Olympus microscope equipped with a 40× water-immersion lens, differential interference contrast, and infrared optical filter. Somatic whole-cell recordings were obtained from putative DRn 5-HT neurons with patch electrodes (3–5 mΩ) filled with a solution containing (in mm): 120 potassium gluconate; 10 KCl; 10 Na_2_-phosphocreatine; 10 HEPES; 1 MgCl_2_; 1 EGTA; 2 Na_2_-ATP; and 0.25 Na-GTP, pH 7.3, osmolarity 280–290 mOsmol. DRn 5-HT neurons were identified by their distinct electrical properties, which include slow firing activity induced by suprathreshold membrane depolarization, large afterhyperpolarization, and membrane hyperpolarization induced by 5-HT_1A_ receptor agonist as previously described (Haj-Dahmane, 2001; Geddes et al. 2015).

All recordings were performed from putative 5-HT neurons located in the dorsomedial subdivisions of the DRn. Excitatory postsynaptic currents (EPSCs) were evoked with single square-pulses (duration, 100–200 µs) delivered at 0.1 Hz with patch pipettes (2–3 mΩ) filled with artificial CSF (ACSF) and placed (50–100 µm) dorsolateral to the recording sites. In some experiments, to assess the change in paired-pulse ratio (PPR), pairs of EPSCs were evoked with an interstimulus interval of 30 ms. The intensity of the stimulus was adjusted to evoke 75% of the maximal amplitude of EPSCs. AMPAR-mediated EPSCs were recorded from neurons voltage clamped at –70 mV in the presence of GABA_A_ and glycine receptor antagonists picrotoxin (100 µM) and strychnine (20 µM), respectively. Membrane currents were amplified with an Axoclamp 2B or Multiclamp 700B amplifier (Molecular Devices). Membrane currents were filtered at 3 kHz, digitized at 20 kHz with Digidata 1440, and acquired using pClamp 10 software (Molecular Devices). The cell input resistance and access resistance (10–20 mΩ) were monitored throughout the experiment using 5-mV hyperpolarizing voltage steps (500-ms duration). Recordings were discarded when the input and series resistance changed by >10% to 20%.

**Figure 1.**
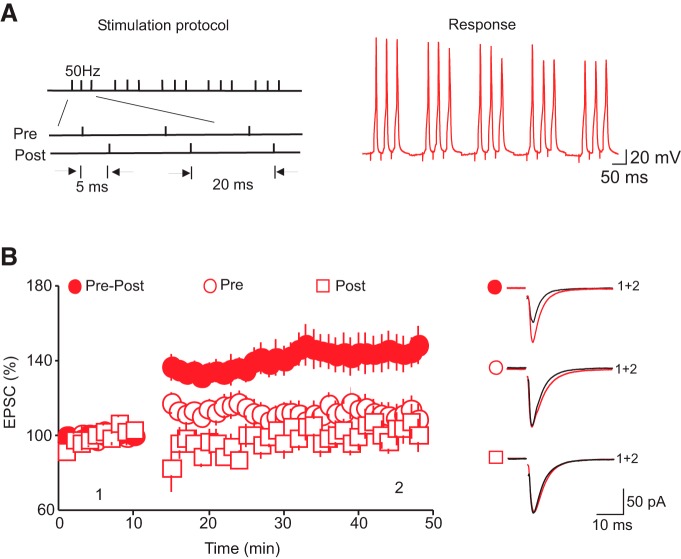
Pairing presynaptic stimulation with bAPs induces tLTP of AMPAR-EPSCs. ***A***, Stimulation protocol (left) and voltage response (right) used to induce tLTP. ***B***, Summary graph of the time course and the magnitude of the potentiation of AMPAR-EPSCs induced by pairing presynaptic stimulations with bAPs (•, *n* = 26), presynaptic stimulation alone (O, *n* = 10), and bAPs (□, *n* = 10). Right graph illustrates averaged AMPAR-EPSC traces taken at the time point indicated in the left graph. Note that the induction of the LTP requires pairing of pre- and postsynaptic stimulations.

To examine whether glutamate synapses onto DRn 5-HT neurons exhibit activity-dependent change in strength, we used an induction protocol that consisted of pairing a train of five bursts of presynaptic stimulation with back-propagating action potentials (bAPs) delivered at 5 Hz. Each burst was composed of three presynaptic stimuli (50 Hz) paired with three bAPs (50 Hz) with a delay of 5–10 ms (Fig. 1*A*). Action potentials were evoked by injection of depolarizing somatic current (1.5–2 nA, 2-ms duration) in current clamp mode. After obtaining a stable recording of AMPAR-EPSCs for at least 10 min, the recordings of DRn 5-HT neurons were switched to current clamp mode, and a total of 20 trains were administered at 0.1 Hz.

### Data analysis

EPSCs were analyzed using Clampfit 10.2 software (Molecular Devices). The amplitude of EPSCs was determined by measuring the average current during a 2-ms time window at the peak of each EPSC and subtracting from the baseline current determined during a 5-ms time window before the stimulus artifact. All EPSC amplitudes were normalized to the mean baseline amplitude recorded for at least 10 min before administration of the pairing protocol. For paired pulse experiments, pairs of stimuli were given at 30-ms intervals. The paired pulse ratios (PPR = EPSC_2_/EPSC_1_) were averaged for at least 60 trials before and 30–40 min after administration of the STDP protocol. To determine the coefficient of variation (CV), the SD and the mean amplitude of EPSCs were calculated for at least 60 consecutive trials before and during the tLTP. The CV was then determined by the following ratio: SD/EPSC mean amplitude. Statistical analysis was performed using Origin 8.0 software (Microcal Software). The results in the text and figures are expressed as means ± SEM. Statistical comparisons were conducted using the Student’s paired *t* test for within-group comparisons and the independent *t* test for comparisons between groups. Statistical significance was set at *p* < 0.05.

### Chemicals

Most chemicals were obtained from Thermo Fisher Scientific. 1,2-Bis(2aminophenoxy)ethane-N,N,N′,N′-tetraacetic acid (BAPTA), *S*-nitroso-*N*-acetyl-dl-penicillamine (SNSP), *N*_ω_-nitro-l-arginine methyl ester hydrochloride (L-NAME), 2-(4-carboxyphenyl)-4,4,5,5-tetramethylimidazoline-1-oxyl-3-oxide (carboxy-PTIO), and 8-(4-chloorophenylthio)-guanosine 3′,5′-cyclic monophosphate (pCPT-cGMP) were purchased from Sigma-Aldrich. Picrotoxin, strychnine, d-(-)-2-amino-5-phosphonopentanic acid (D-AP5), 1H-(1,2,4)oxadiazolo(4,3-a)quinaxalin-1-one (ODQ), and 1-naphthyl acetyl spermine trihydrochloride (Napsm) were obtained from Tocris Biosciences.

## Results

### Glutamate synapses onto DRn 5-HT neurons exhibit tLTP

Activity-dependent change in synaptic strength is a fundamental neuronal mechanism involved in learning, memory, and behavioral adaptation (Bliss and Lomo, 1973; Malenka and Bear, 2004). Long-term potentiation (LTP) and long-term depression (LTD), the two best-studied forms of synaptic plasticity, have been reported at both excitatory and inhibitory synapses in several brain regions (Huganir and Nicoll, 2013). To investigate whether glutamate synapses in the DRn undergo activity-dependent alterations in strength, we performed whole-cell recordings from putative DRn 5-HT neurons. A monopolar glass stimulating patch electrode was positioned close to the recorded neuron, and AMPAR-EPSCs were evoked at 0.1 Hz. We applied a plasticity stimulation protocol that consisted of repetitive pairing of presynaptic stimulation with postsynaptic spiking of DRn 5-HT neurons with a positive delay of 5–10 ms (Fig.1*A*) and found that it induced a robust and sustained potentiation of the amplitude of AMPAR-EPSCs (158.36 ± 6.35% of baseline; *n* = 26; *p* < 0.01, Fig. 1*B*). This spike-timing–dependent potentiation (tLTP) lasted for the duration of the recordings (>50 min). To test whether the coincident activity of both pre- and postsynaptic neurons is necessary for the tLTP induction, we first examined the effect of repetitive presynaptic stimulation alone and found that it failed to potentiate the amplitude of AMPAR-EPSCs (112.17 ± 7.39% of baseline, *n* = 10, *p* > 0.05, Fig. 1*B*). Next, we applied only the postsynaptic component of our plasticity protocol (i.e., repetitive firing of postsynaptic 5-HT neurons) and found that this manipulation alone did not induce a significant potentiation of AMPAR-EPSCs (103.04 ± 8% of baseline, *n* = 10, *p* > 0.05, Fig. 1*B*). Collectively, these results indicate that pairing pre- and postsynaptic activity is required for the induction of tLTP of glutamate synapses onto putative DRn 5-HT neurons.

### NMDAR activation is not required for tLTP induction in the DRn

**Figure 2.**
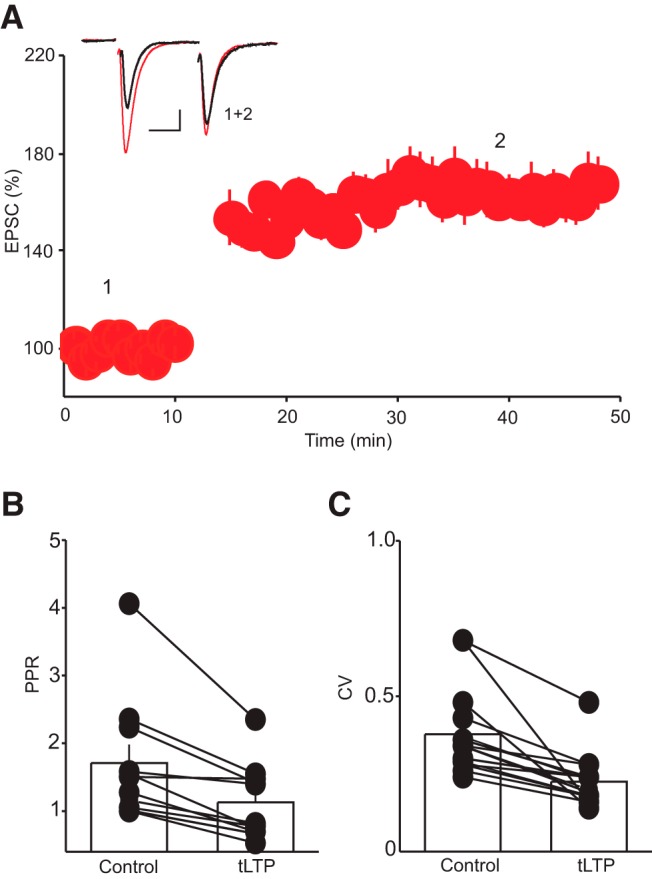
Decrease in PPR and CV indicate a presynaptic expression of tLTP. ***A***, Summary graph of tLTP assessed using pairs of stimuli. ***B***, Histogram summary of the average PPR (EPSC_2_/EPSC_1_) obtained before and during the tLTP. Inset depicts superimposed EPSC traces evoked by paired-pulse stimulation taken at the time point indicated by number. Scale bars: 50 pA, 20 ms. ***C***, Histogram summary of the average CV obtained before and during the tLTP. Note that the tLTP is associated with a significant decrease in PPR (*, p < 0.05, *n* = 11) and CV (*, p < 0.05, *n* = 15).

Canonical tLTP of glutamate synapses is mediated by NMDAR-dependent mechanisms (Feldman, 2012) that involve an increase in the number or single-channel conductance of AMPARs (Malinow and Malenka, 2002; Feldman, 2012). To determine the features of the tLTP of glutamate synapses in the DRn, we first examined the locus of tLTP expression by monitoring the PPR, as determined by the ratio of EPSC_2_/EPSC_1_, and the CV of AMPAR-EPSCs before and during tLTP (Fig. 2*A*), two metrics that report alterations in presynaptic function. We found that the tLTP was consistently associated with a significant decrease in both the PPR (control, 1.18 ± 0.05; tLTP, 0.77 ± 0.03, *n* = 11, *p* < 0.05, Fig. 2*B*) and CV (CV control, 0.38 ± 0.03, CV tLTP, 0.22 ± 0.02, *p* < 0.05, *n* = 15, Fig. 2*C*), indicating that it is mediated by an increase in glutamate release.

**Figure 3.**
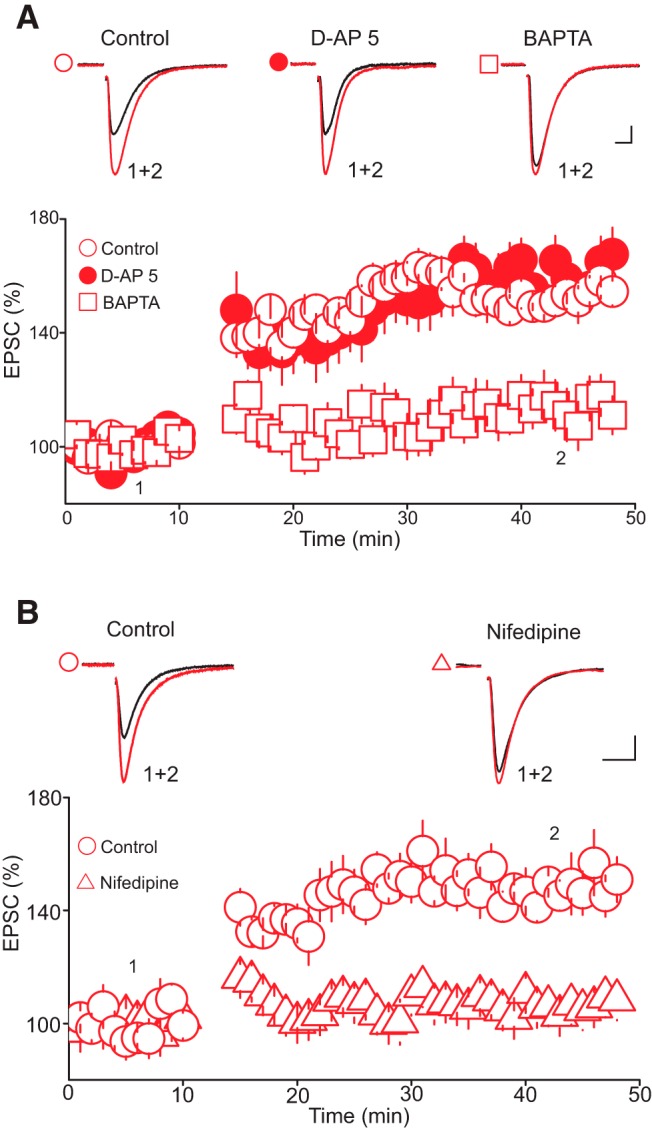
The tLTP requires a rise in postsynaptic intracellular Ca^2+^ but not the activation of NMDA receptors. ***A***, Buffering postsynaptic intracellular Ca^2+^, but not blockade of NMDA receptors, abolishes the tLTP. Lower panel is a summary graph of tLTP obtained in control condition (O, *n* = 11), in the presence of D-AP 5 (•, 50 µM, *n* = 10), and with intracellular solution containing BAPTA (□, 20 mM, *n* = 10). Upper panel illustrates superimposed averaged AMPAR-EPSC traces taken at time points indicated in lower graph. Scale bars: 20 pA, 5 ms. ***B***, Blockade of voltage-dependent Ca^2+^ channels abolishes tLTP. Lower panel is a summary graph of the time course and magnitude of tLTP obtained in control (O, *n* = 10) and in the presence of nifidepine (Δ, 20 µM, *n* = 11). Upper panel illustrates superimposed AMPAR-EPSC traces taken before and during the tLTP in control (left traces) and in the presence of nifidepine (right traces). Scale bars: 25 pA, 10 ms.

We next examined whether the induction of the tLTP requires an increase in postsynaptic intracellular calcium (Ca^2+^). To that end, we assessed the impact of buffering intracellular Ca^2+^ with the fast calcium chelator BAPTA on the magnitude and time course of the tLTP. Buffering postsynaptic intracellular Ca^2+^ with BAPTA (20 mm in the recording pipette) profoundly reduced the magnitude of the tLTP (tLTP control, 152.75 ± 6.75% of baseline, tLTP BAPTA, 111.75 ± 7.65% of baseline, *n* = 10, *p* < 0.05 vs. control, Fig. 3*A*), indicating that an increase in postsynaptic intracellular Ca^2+^ is required for the induction of the tLTP. We next determined the contribution of Ca^2+^ influx through NMDA receptors by examining the effect of NMDA receptor antagonist D-AP5 (50 µM) on the tLTP. Unexpectedly, blockade of NMDARs with D-AP5 did not prevent the induction of tLTP. Indeed, the magnitude and time course of the tLTP obtained in the presence of D-AP5 were indistinguishable from those obtained in control condition (tLTP control, 152.75 ± 6.75% of baseline; tLTP D-AP5, 160.06 ± 8.75% of baseline, *n* = 10, *p* > 0.05 vs. control, Fig. 3*A*). The tLTP obtained in the presence of D-AP5 was also associated with a decrease in PPR (PPR control, 1.28 ± 0.04; tLTP, 0.83 ± 0.06, *p* < 0.05, *n* = 10) and CV (CV control, 0.34 ± 0.05; CV tLTP, 0.21 ± 0.03, *p* < 0.05, *n* = 10). In the search for an alternative source of Ca^2+^ entry during tLTP induction, we next blocked voltage-dependent Ca^2+^ channels with nifidepine (20 µM) and found that it abolished tLTP (tLTP control, 147.89 ± 6.76% of baseline; tLTP nifidepine, 106.23 ± 5.85% of baseline, *p* < 0.05 vs. control, *n* = 11, Fig 3*B*). Collectively, these results indicate that Ca^2+^ influx through voltage-dependent Ca^2+^ channels, but not NMDARs, is necessary for the induction of tLTP in DRn 5-HT neurons.

**Figure 4.**
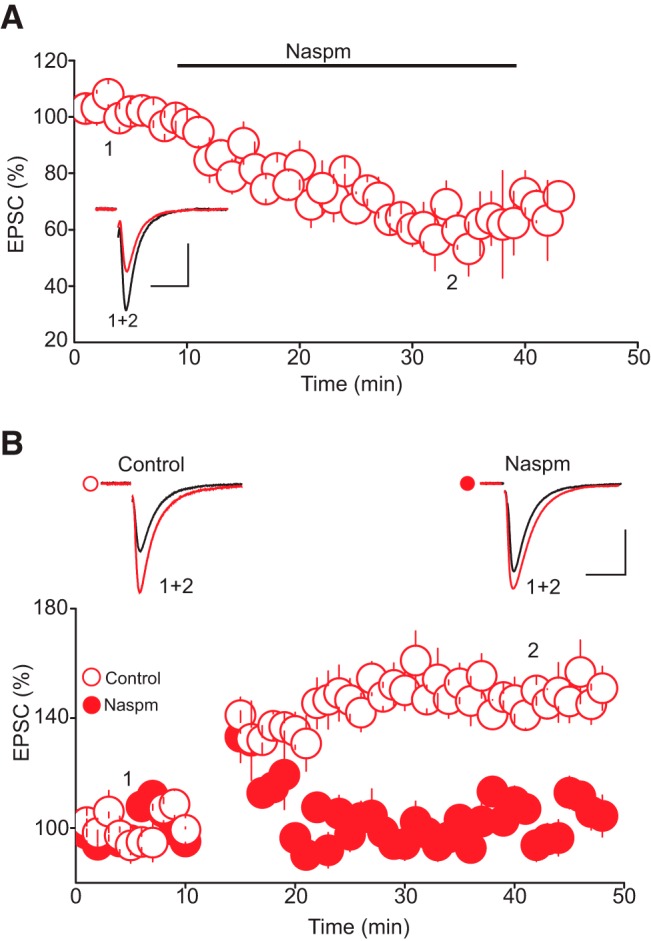
Activation of GluA2-lacking AMPARs is required for tLTP induction. ***A***, GluA2-lacking AMPARs significantly contribute to the baseline amplitude of AMPAR-EPSCs. Lower panel depicts the effect of the selective GluA2-lacking AMPAR antagonist Napsm (30 µM, *n* = 8) on the baseline amplitude of AMPAR-EPSCs. Upper graph is superimposed AMPAR-EPSC traces taken during the course of the experiment as indicated by numbers in lower panel. ***B***, Blockade of GluA2-lacking AMPARs abolishes tLTP. Lower graph is a summary of the time course and magnitude of tLTP obtained in control condition (O, *n* = 8) and in the presence of Napsm (30 µM, •, *n* = 8). Upper graph depicts superimposed AMPAR-EPSC traces collected before and during tLTP in control condition (left traces) and in the presence of NASPAM (right traces). Scale bars: 50 pA, 10 ms.

The finding that activation of NMDARs is not required for tLTP induction, in combination with the observation that neither pre- nor postsynaptic stimulation alone reliably induces tLTP, suggests the presence of a synaptic source of Ca^2+^ during the induction phase of tLTP in addition to that afforded by voltage-dependent Ca^2+^ channels. We thus reasoned that Ca^2+^ influx through Ca^2+^-permeable AMPARs (i.e., GluA2-lacking AMPARs) during synaptic stimulation might contribute to the increase in intracellular Ca^2+^ signal (Jia et al. 1996; Wiltgen et al. 2010) necessary for tLTP induction. To begin testing this idea, we first determined the overall contribution of GluA2-lacking AMPARs to glutamatergic transmission onto DRn 5-HT neurons. To that end, we examined the effect of the selective GluA2-lacking AMPAR antagonist Napsm (30 µM) and found that it reduced the amplitude of AMPAR-EPSCs to 63.05 ± 10.35% of baseline (*n* = 8, *p* < 0.05 vs. baseline, Fig. 4*A*). This finding indicates that GluA2-lacking AMPARs contribute to a significant fraction of AMPAR-EPSPs onto 5-HT neurons and may thus act as a Ca^2+^ source during tLTP (Camiré and Topolnik, 2014). Therefore, we next directly tested the impact of Napsm on the magnitude and time course of tLTP. As illustrated in Fig. 2*D*, treatment of slices with Napsm (50 µM) prevented the induction of the tLTP (control tLTP, 147.95 ± 7.5% of baseline; Napsm tLTP, 107.83 ± 6.62% of baseline, *p* < 0.05 vs. control, *n* = 8, Fig. 4*D*). These results indicate that Ca^2+^ influx through activation of GluA2-lacking AMPARs contributes to tLTP induction.

### NO mediates tLTP of glutamate synapses onto DRn 5-HT neurons

**Figure 5.**
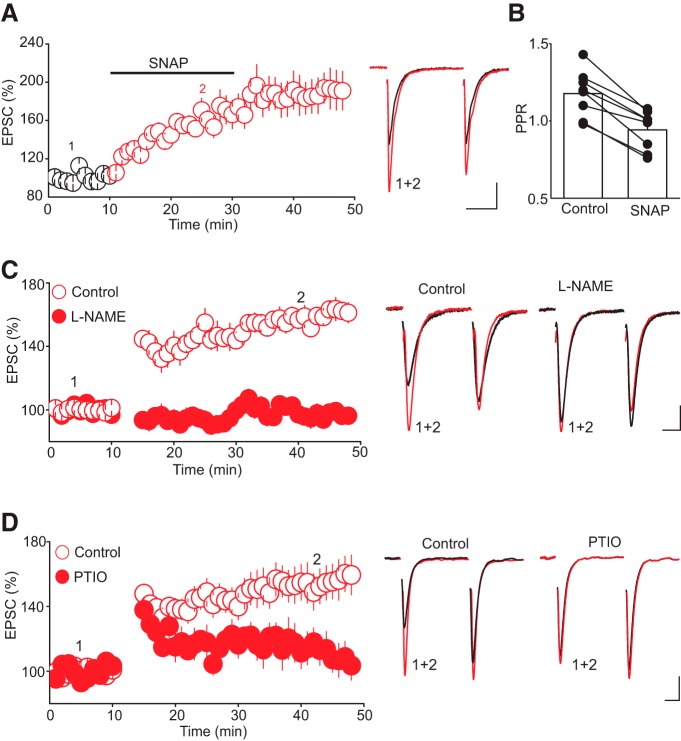
Nitric oxide signaling mediates tLTP. ***A***, The nitric oxide donor SNAP increases AMPAR-EPSC amplitude by enhancing glutamate release. Left panel illustrates summary graph of the effect of SNAP (200 µM, *n* = 8) on the amplitude of AMPAR-EPSCs. Middle panel illustrates superimposed average pairs of EPSC traces collected before and during SNAP application. ***B***, Summary histogram of changes in PPR of AMPAR-EPSCs obtained in control and in the presence of SNAP. Note that SNAP (200 µM) significantly (*, p < 0.05, *n* = 8) reduced the PPR. ***C***, Nitric oxide synthase inhibitor L-NAME abolishes tLTP. Left graph illustrates summary graph of tLTP obtained in control condition (O, *n* = 14) and in slices pretreated with L-NAME (100 µM, •, *n* = 14). Right panel represents sample average AMPAR-EPSC traces taken during the experiment as depicted by numbers in the left graph. ***D***, The NO scavenger PTIO prevents tLTP. Left panel illustrates the time course and magnitude of tLTP obtained in control (O, *n* = 8) and in the presence of NO scavenger PTIO (100 µM, •, *n* = 8). Left graph is superimposed AMPAR-EPSC traces taken during the course of experiment as indicated by number in the left graph. Scale bars: 25 pA, 10 ms.

Collectively, our results indicate that tLTP in 5-HT neurons is induced postsynaptically, but expressed presynaptically by means of a robust increase in glutamate release. These results thus raise the possibility that tLTP expression involves retrograde signaling. Despite some initial controversial findings, the role of retrograde signaling in mediating several types of plasticity has been demonstrated for several synapses in the brain (Mu and Poo, 2006; Fino et al. 2009). Because of the remarkably high level of expression of neuronal nitric oxide synthase (nNOS, a key enzyme for NO synthesis) in DRn 5-HT neurons (Xu and Hökfelt, 1997; Simpson et al. 2003), we hypothesized that NO was the retrograde messenger mediating tLTP. To test this hypothesis, we first examined whether NO donors could mimic the tLTP. We found that bath application of SNAP (200 µM) robustly increased the amplitude of AMPAR-EPSCs (188. 49 ± 19.02% of baseline, *n* = 8, *p* < 0.05, Fig. 5*A*). This effect was accompanied by a significant decrease in PPR (PPR control, 1.18 ± 0.05; PPR SNAP, 0.94 ± 0.04, *n* = 8, *p* < 0.05, Fig. 5*B*), indicating that increasing NO signaling potentiates glutamatergic synaptic transmission onto DRn 5-HT neurons by increasing glutamate release, thereby mimicking tLTP.

We next examined whether reducing NO levels using two distinct strategies prevents tLTP induction. First, we treated DRn slices with L-NAME (100 µm), an inhibitor of nNOS, and found that it abolished tLTP (tLTP interleaved controls, 159.42 ± 5.5% of baseline; tLTP L-NAME, 97.27 ± 5.39% of baseline; *n* = 14, *p* < 0.05, Fig. 5*C*). Second, we treated DRn slices with the NO scavenger PTIO (100 µm) and found that it also blocked the induction of tLTP (tLTP control, 1.55.05 ± 6.5% of baseline; tLTP PTIO, 101.75 ± 5.6% of baseline; *n* = 8, *p* < 0.05 vs. control, Fig. 5*D*). Thus, these results indicate that an increase in enzymatically driven NO is necessary for triggering tLTP in DRn 5-HT neurons.

### NO-cGMP–dependent activation of PKG mediates tLTP

**Figure 6.**
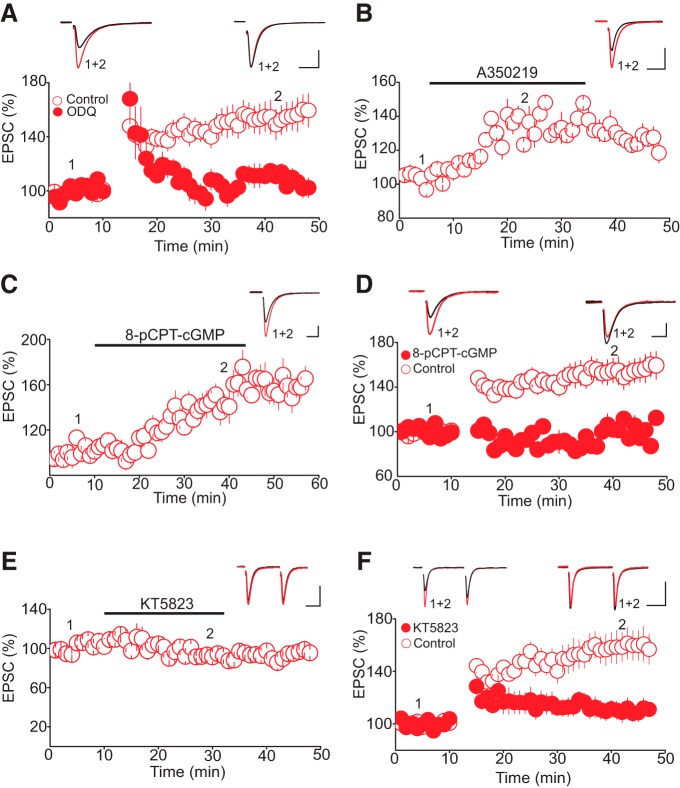
Activation of cGMP-PKG pathway mediates tLTP. ***A***, Inhibition of sGC blocks tLTP. Lower graph is a summary of tLTP obtained in control condition (O, *n* = 10) and in slices treated with the sGC inhibitor ODQ (100 µM, •, *n* = 10). Upper graph illustrates superimposed AMPAR-EPSC traces taken before and during tLTP as indicated by numbers in lower graph. ***B***, Activation of sGC mimics tLTP. Lower panel depict a summary of the potentiation of AMPAR-EPSC induced by the selective sGC activator A350219 (100 µM, *n* = 7). Upper graph is superimposed AMPAR-EPSC traces taken before and during A350219 administration. ***C***, The membrane permeable cGMP analog 8-pCPT-cGMP mimics tLTP. Lower graph illustrates the averaged potentiation of AMPAR-EPSCs induced by 8-pCPT-cGMP (100 µM, *n* = 7). Upper graph is superimposed traces of AMPAR-EPSCs taken before and during administration of 8-pCPT-cGMP. ***D***, The membrane permeable cGMP analog 8-pCPT-cGMP occludes tLTP. Lower panel is a summary graph of the tLTP obtained in control condition (O, *n* = 8) and in the presence of 8-pCPT-cGMP (100 µM, •, *n* = 8). Upper graph depicts AMPAR-EPSCs traces taken at the time points indicated by numbers in lower graph. ***E***, Inhibition of PKG does not alter the baseline amplitude of AMPAR-EPSCs. Lower panel is a summary graph of the effect of KY5823 (1 µM, *n* = 6) on the amplitude of AMPAR-EPSCs. Upper graph illustrates sample AMPAR-EPSC traces taken during the course of the experiments as indicated by numbers in the lower panel. ***F***, Inhibition of PKG abolishes tLTP. Lower panel illustrates a summary of tLTP obtained in control (O, *n* = 8) and in slices treated with the PKG inhibitor KT5823 (1 µM, •, *n* = 5). Scale bars: 50 pA, 20 ms.

The physiologic effects of NO are generally signaled through the activation of soluble guanylate cyclase (sGC), leading to an increase in cGMP production and stimulation of cGMP-dependent protein kinases (PKG; el-Husseini et al. 1995; Francis et al. 2010). Activation of this signaling cascade mediates several forms of synaptic plasticity and increases glutamate and GABA release at central synapses (Boulton et al. 1995; Nugent et al. 2009; Pigott and Garthwaite, 2016). Therefore, we wondered whether activation of the cGMP–PKG pathway could mediate tLTP of glutamate synapses in the DRn. To test this notion, we first examined the impact of sGC inhibition on the induction of tLTP. As illustrated in Fig. 6*A*, we found that in brainstem slices treated with the sGC inhibitor ODQ (100 µm), the pairing protocol triggered only a transient potentiation of AMPAR-EPSCs. The amplitude of AMPAR-EPSCs recovered to baseline levels within ∼10 min after pairing (tLTP control, 154.29 ± 3.94% of baseline; tLTP ODQ, 108.05 ± 5.94% of baseline, *n* = 10, *p* > 0.05 vs. baseline; *p* < 0.05 vs. control, Fig. 6*A*), indicating that tLTP requires the activation of sGC. To further examine the involvement of sGC in mediating tLTP, we next tested whether activation of sGC could potentiate AMPAR-EPSCs. Bath application of the selective sGC activator A350219 (100 µm) significantly increased the amplitude of AMPAR-EPSCs (133.57 ± 8.84% of baseline, *n* = 7, *p* < 0.05, Fig. 6*B*). The ability of sGC inhibitors and activators to prevent and mimic tLTP, respectively, suggests that activation of sGC is necessary for tLTP induction at glutamate synapses onto DRn 5-HT neurons.

If activation of the sGC and the subsequent increase in the cGMP levels were to mediate tLTP, administration of membrane-permeable cGMP analogs should mimic and occlude tLTP. Indeed, we found that bath application of 8-pCPT-cGMP (100 µm), a membrane-permeable cGMP analog, potentiated the amplitude of AMPAR-EPSCs (158.89 ± 9.35% of baseline, *p* < 0.05 vs. baseline, *n* = 7, Fig. 6*C*). Importantly, in slices pretreated with 8-pCPT-cGMP (100 µm), our pairing protocol failed to induce tLTP (tLTP control, 155.24 ± 3.38% of baseline; tLTP 8-pCPT-cGMP, 101.12 ± 8.23% of baseline, *n* = 8, *p* < 0.05 vs. control, Fig. 6*D*), indicating that treatment with the cGMP analog occludes tLTP. Collectively, these results indicate that NO-mediated activation of sGC and subsequent increase in cGMP are required for induction of tLTP.

To test whether NO-cGMP signaling mediates tLTP by activating PKG, we examined the impact of the selective PKG inhibitor KT5823 on tLTP. Whereas administration of KT5823 (1 µm), which did not alter the baseline amplitude of AMPAR-EPSCs (95.60 ± 5.9% of baseline, *p* > 0.05, *n* = 6, Fig. 6*E*), blocked tLTP (tLTP interleaved controls, 146.85 ± 5.8% of baseline; tLTP KT5826, 105.42 ± 6.5% of baseline, *p* < 0.05 vs. control, *n* = 8, Fig. 6*F*). Lastly, treatment with KT5826 prevented the potentiation of AMPAR-EPSC induced by 8-pCPT-cGMP (100 µm; 103.56 ± 6.8% of baseline, *n* = 5, data not shown). Collectively, these results indicate that the increase in NO induced by a pairing protocol leads to the activation of sGC and PKG signaling cascade that mediates tLTP of glutamate synapses in the DRn.

## Discussion

The results of the present study demonstrate that glutamate synapses onto DRn 5-HT neurons are plastic and exhibit tLTP. This form of LTP is initiated by a rise in postsynaptic intracellular Ca^2+^ and expressed by a persistent increase in the probability of glutamate release. Importantly, our results show that the Ca^2+^ signals required for tLTP induction are mediated by the activation of voltage-dependent calcium channels (VDCCs) and GluA2-lacking AMPARs, but not NMDARs. In addition, we show that the presynaptic expression of tLTP is mediated by the NO/cGMP signaling cascade. As such, this study provides direct evidence that correlated pre- and postsynaptic activity within the DRn strengthens glutamate synapses onto 5-HT neurons. It also unravels a previously unsuspected role of NO/cGMP signaling in controlling synaptic plasticity in the DRn.

At most glutamate synapses studied, repetitive and correlated pre- and postsynaptic action potentials induce an NMDAR-dependent tLTP (Markram et al. 1997; Feldman, 2012). The required temporal association (i.e., pre- before post-) is largely attributed to the coincidence detection feature of postsynaptic NMDARs (Song et al. 2000; Rubin et al. 2005), leading to an increase in intracellular Ca^2+^ (Holbro et al. 2010) and activation of downstream biochemical cascades mediating the tLTP. Unexpectedly, at glutamate synapses onto DRn 5-HT neurons, we found that although tLTP requires a rise in postsynaptic intracellular Ca^2+^, it is independent of NMDAR activation. Indeed, a robust tLTP can be elicited in the presence of NMDAR blocker, suggesting that Ca^2+^ influx through VDCCs and/or calcium-permeable AMPARs (CP-AMPARs) signals the induction of tLTP. Consistent with this notion, we show that blocking VDCCs abolishes the tLTP. Similarly, no tLTP could be induced in the presence of CP-AMPAR antagonist, indicating that joint activation of VDCCs and CP-AMPARs is required for tLTP of glutamate synapses onto DRn 5-HT neurons. Such a conclusion is in agreement with previous reports of CP-AMPAR– and VDCC-dependent LTP of synapses in other brain areas (Galvan et al. 2008; Hainmüller et al. 2014).

The finding that coincident pre- and postsynaptic activity is required for tLTP induction in DRn 5-HT neurons demonstrates its associative nature and indicates that the increase in intracellular Ca^2+^ evoked by activation of either CP-AMPAR or bAPs alone is not sufficient to trigger the biochemical cascade mediating tLTP. It thus appears that postsynaptic intracellular Ca^2+^ of sufficient magnitude to reach threshold for tLTP induction is achieved only when activation of CP-AMPARs is paired with bAPs. This finding is consistent with previous studies of glutamate synapses in other brain areas showing that blockade of CP-AMPARs significantly reduces the magnitude of spine Ca^2+^ signals during pairing (Holbro et al. 2010) and prevents Hebbian LTP (Galvan et al. 2008; Holbro et al. 2010).

We have shown that the tLTP of glutamate synapses onto DRn 5-HT neurons is initiated by a rise in postsynaptic intracellular Ca^2+^ but is expressed as a persistent increase in glutamate release. This is supported by the finding that tLTP was invariably accompanied by an increase in the probability of neurotransmitter release, as inferred from a decrease in both PPR and CV. More importantly, because pharmacological manipulations that enhance NO synthesis mimicked and occluded tLTP and inhibition of NO synthesis prevented tLTP induction, we concluded that the presynaptic expression of tLTP is mediated by NO. This conclusion is in agreement with the well-established role of NO as a retrograde messenger at central synapses (Brenman and Bredt, 1997; Garthwaite, 2016) mediating several form of synaptic plasticity (Boulton et al. 1995; Hardingham et al. 2013), including presynaptic LTP of glutamate (Szabo et al. 2012; Pigott and Garthwaite, 2016) and GABA synapses (Nugent et al. 2009) in other brain areas. It is generally thought that nNOS is mainly activated by Ca^2+^ influx through postsynaptic NMDARs (Holscher, 1997). This mode of coupling is facilitated by the distribution of nNOS in the postsynaptic density tethered to NMDARs (Brenman et al. 1996). Here, we show that Ca^2+^ influx induced by joint activation of CP-AMPARs and VDCC mediates the activation of nNOS and triggers the synthesis of NO. Such findings are consistent with recent studies demonstrating that nNOS can be activated by Ca^2+^ influx through VDCCs (Pigott and Garthwaite, 2016) and CP-AMPARs (Szabo et al. 2012), indicating that the source of intracellular Ca^2+^ involved in the activation of nNOS is more diverse than initially thought.

NO signaling can enhance glutamate release and induce tLTP by nitrosothiol generation in a number of proteins of the release machinery (Meffert et al. 1996) or by activation of the presynaptic sGC/cGMP pathway (Neitz et al. 2011; Eguchi et al. 2012). Here, we find that pharmacological manipulations that increase cGMP level potentiate glutamate synapses and occlude tLTP. In contrast, inhibition of sGC and PKG abolish tLTP induction, indicating that NO signals the tLTP of glutamate synapses onto DRn 5-HT neurons via activation of cGMP–PKG signaling cascade. The involvement of this canonical signaling pathway of NO is consistent with several studies showing that activation of the cGMP–PKG pathway increases the probability of neurotransmitter release (Arancio et al. 1995; Hardingham et al. 2013). Importantly, activation of this signaling cascade has been shown to mediate the induction and maintenance of NO-mediated presynaptic LTP of glutamatergic (Zhuo et al. 1994; Liu et al. 2003; Lange et al. 2012) and GABAergic (Nugent et al. 2009) synapses in other brain areas.

The conclusion that NO signaling gates tLTP of glutamate synapses onto DRn 5-HT neurons has established a functional role of the high expression of nNOS in the DRn neurons (Xu and Hökfelt, 1997; Simpson et al. 2003), including 5-HT neurons (Simpson et al. 2003). The involvement of NO is also in agreement with numerous studies showing that NO signaling in the DRn controls a plethora of physiologic functions, including arousal (Monti et al. 1999) and stress homeostasis (Okere and Waterhouse, 2006). Thus, exposure to various stressors has been shown to stimulate nNOS-expressing neurons in the DRn (Krukoff and Khalili, 1997; Okere and Waterhouse, 2006). Moreover, activation of NO signaling in the DRn exerts anorexigenic effects (Currie et al. 2011) and enhances anxiety-like behaviors (Grahn et al. 2000), whereas inhibition of NO signaling within the DRn increases arousal, inhibits anxiety-like behaviors, and elicits antidepressant like effects, at least in part, through modulation of 5-HT neurons (Spiacci et al. 2008; Miguel et al. 2010). The present finding that NO signaling modulates the strength and plasticity of glutamate synapses onto DRn 5-HT neurons further supports functional interaction between NO and the 5-HT system and provides a potential cellular mechanism by which NO signaling regulates the function of 5-HT neurons and stress-related behaviors.

Early studies have suggested that DRn 5-HT neurons are mainly involved in the regulation of general homeostatic functions such as the sleep-waking cycle (Jacobs and Fornal, 1999), locomotion (Jacobs and Fornal, 1997), emotion, and stress homeostasis (Cools et al. 2008). However, more recently, numerous studies have extended the role of these neurons to include modulation of behavioral tasks that require associative learning. Results from numerous investigations of motivated behaviors have shown that DRn 5-HT neurons exert potent effects on behavioral actions to either gain rewards or avoid punishments (Liu and Ikemoto, 2007; Nakamura et al. 2008). Consequently, during motivated behavioral tasks, DRn 5-HT neurons respond to reward-related events by gradual tonic change in their activity lasting throughout multiple phases of behavioral tasks, indicating that the activity of these neurons encodes sustained aspects of motivated behaviors (Liu et al. 2014; Cohen et al. 2015). Similarly, in learned helplessness behaviors, exposure to inescapable aversive stimuli (IS) induces long-lasting changes of the response of DRn 5-HT neurons to IS (Grahn et al. 1999, 2000). This tonic change in the activity of DRn 5-HT neurons encode the impaired escape behavior and the acquisition of conditioned fear (Grahn et al. 2000). Collectively, these studies have led to the notion that tonic change in the electrical activity of DRn 5-HT neurons encodes value representation of the stimuli in associative learning (Nakamura and Wong-Lin, 2014; Dayan and Huys, 2015). The present finding that glutamate synapses onto DRn 5-HT neurons undergo associative plasticity (i.e., tLTP) provides a synaptic correlate to these behaviors and additional support for the role of DRn 5-HT neurons in associative learning. Importantly, studies of learned helplessness behaviors have shown that the increased response of DRn 5-HT neurons to IS exposure involves an LTP-like process of excitatory inputs to the DRn that requires activation of NO signaling (Grahn et al. 2000). Consequently, it is tempting to speculate that the NO-mediated tLTP reported in the present study could be a cellular mechanism mediating the persistent change in the activity of DRn 5-HT neurons induced by repetitive exposure to reward or aversive-related stimuli. However, additional studies are required to define the precise role of LTP of glutamate synapses onto DRn 5-HT neurons in encoding goal-directed behaviors, including conditioned fear.

## Synthesis

Reviewing Editor: Pablo Castillo, Albert Einstein College of Medicine Decisions are customarily a result of the Reviewing Editor and the peer reviewers coming together and discussing their recommendations until a consensus is reached. When revisions are invited, a fact-based synthesis statement explaining their decision and outlining what is needed to prepare a revision will be listed below. The following reviewer(s) agreed to reveal their identity: Karri Lamsa, Christopher Ford.

Your manuscript has been evaluated by two reviewers. Here is the synthesis of their online discussion. They agree that describing use-dependent long-term plasticity at excitatory inputs onto neurons of the dorsal raphe nucleus is novel. They think the experiments are well designed, the results are convincing, and the identified mechanism of synaptic plasticity is exciting. As you will see below, they also raised a number of concerns, highlighting the identity of the recorded cells (or lack thereof) and the high dose of nifedipine used in your experiments.

---------------------------------------------

REVIEWER #1

I have two major concerns, both about the experimental design (please see below). In addition, although the manuscript in general reads well it has various typos and many small errors in it. Finally, some relevant references are missing.

Major comments:

1) The NO donor experiments have continuous wash-in of SNAP. Given that this experiment aims to replicate the LTP induction, it would be more relevant to apply SNPA only briefly, wash-it out and then follow the EPSC for at least 30 min.

2) Nifedipine concentration: It seems that 200 microM nifedipine was used. This sound very high compared to commonly used 10-20 microM. Please clarify (with references) selectivity of nifedipine in this concentration. Nifedipine has been reported to inhibit e.g. cytochrome P450 (Katoh et al. 2000).

Minor comments:

-The abstract simply ignores the significant result on the contribution of VGCC in the LTP (cf. lines 235-237 in results).

-Line 38 (and other places similarly stating "GluA2-lacking..."). Please do not use GluA2- lacking, since CP-AMPARs can also emerge from the unedited GluA2 subunit.

-Line 211: Please state here (and not only in the methods) how PPR was calculated.

-Lines 228-231: Please report also PPR and CV values for the experiments showing LTP in the presence of APV.

-Lines 242-245: Please add two references here: 1) Enhanced LTP in Mice Deficient in the AMPA Receptor GluR2. Zhengping Jia et al.

doi.org/10.1016/S0896-6273(00)80225-1. 2) A Role for Calcium-Permeable AMPA Receptors in Synaptic Plasticity and Learning. Brian J. Wiltgen et al. doi.org/10.1371/journal.pone.0012818.

-Lines 250-251: Please add references to support this conclusion (with Naspm effect). For instance: Dendritic calcium nonlinearities switch the direction of synaptic plasticity in fast-spiking interneurons. Camire O, Topolnik L. doi: 10.1523/JNEUROSCI.2253-13.2014.

-Figure 1A schematic is confusing: it is unclear what the "50 Hz" (upper right corner) refers to and how does it fit with the "200 ms" interval shown below. I suppose this again one of those several typos in the text. Font in the image is inconsistent, and if the distinct fonts are supposed to indicate something, please state it clearly in the legend.

-Figure 2 title: Please reformulate as "EPSC paired-pulse and coefficient of variation indicate presynaptic site for tLTP expression"

-Figure 1B: (also other figures plotting mean values): since the deviation in the plot shows sem rather than sd, it requires showing n number too.

-Figure 2B: For PPR, please indicate the way it was calculate (2nd/1st)

-Figure legend in line 664: Please state if AP5 was D- or DL. This makes a difference regarding the pharmacological blockade.

Typos:

-line 184: Lon- should be Long-

-line 187: 2015 should be 2013

-line 199: AMPR- should be AMPAR-

-line 542: "2015 should be 2013

-line 659: PR should be "PPR

-line 665: BPATA should be BAPTA

Statistics:

Using t-test is justified, since n numbers are quite high (n=8-15 in the experiments). However, I could not find a statement, whether the data pools were tested to show parametric distribution (e.g. with Shapiro-Wilk test). This would be important to add in Methods.

REVIEWER #2

In the present manuscript the authors examine the mechanisms underlying spike timing dependent plasticity in the dorsal raphe. Through a clear set of experiments they find that spike timing dependent plasticity of glutamatergic inputs can be evoked in the raphe and results in an increase in the probability of glutamate release. They report that LTP induction is dependent upon postsynaptic calcium entry by both L-type calcium channels as well as GluA2-lacking AMPA receptors and results in increased glutamate release via the retrograde signaling by nitric oxide. The experiments are done well, the results are clear and the manuscript is well written. There are only minor corrections required and no further experiments are needed.

1) The dorsal raphe consists of several cell types, which have overlapping electrophysiological characteristics, making it difficult to distinguish serotonergic neurons from other cell types. In the manuscript the authors state that recordings were done from putative 5-HT neurons. What criteria were used to define this population of neurons? If no criteria were used to identify 5-HT cells, the authors should instead state throughout the text only that recordings were done in DRn neurons.

2) In most cases, the figure numbers do not match the result section. The authors need to ensure the figures referenced in the text match the correct figures.

3) The results state that 50 uM NASPM was used but the figure legend states 30 uM.

---------------------------------------------
